# Harnessing the AI/ML in Drug and Biological Products Discovery and Development: The Regulatory Perspective

**DOI:** 10.3390/ph18010047

**Published:** 2025-01-03

**Authors:** Fahimeh Mirakhori, Sarfaraz K. Niazi

**Affiliations:** 1 College of Natural and Mathematics Sciences, University of Maryland, Baltimore County (UMBC), USG, Rockville, MD 20850, USA; fmirakh1@umbc.edu; 2 College of Pharmacy, University of Illinois, Chicago, IL 60612, USA

**Keywords:** artificial intelligence (AI), drug product development, regulatory frameworks, ethical consideration, biologics, cell and gene therapy (CGT), advanced therapy medicinal product (ATMP), advanced manufacturing technology (AMT)

## Abstract

Artificial Intelligence (AI) has the disruptive potential to transform patients’ lives via innovations in pharmaceutical sciences, drug development, clinical trials, and manufacturing. However, it presents significant challenges, ethical concerns, and risks across sectors and societies. AI’s rapid advancement has revealed regulatory gaps as existing public policies struggle to keep pace with the challenges posed by these emerging technologies. The term AI itself has become commonplace to argue that greater “human oversight” for “machine intelligence” is needed to harness the power of this revolutionary technology for both potential and risk management, and hence to call for more practical regulatory guidelines, harmonized frameworks, and effective policies to ensure safety, scalability, data privacy, and governance, transparency, and equitable treatment. In this review paper, we employ a holistic multidisciplinary lens to survey the current regulatory landscape with a synopsis of the FDA workshop perspectives on the use of AI in drug and biological product development. We discuss the promises of responsible data-driven AI, challenges and related practices adopted to overcome limitations, and our practical reflections on regulatory oversight. Finally, the paper outlines a path forward and future opportunities for lawful ethical AI. This review highlights the importance of risk-based regulatory oversight, including diverging regulatory views in the field, in reaching a consensus.

## 1. Introduction

Over recent years, the term “Artificial Intelligence” (AI) has become a household word. It was first introduced in a workshop proposal in 1950, driven by neuro-robotic and brain mechanisms envisioned for tomography image analysis [1]. In the 1980s, Fletcher and Doi at the University of Chicago suggested the potential applicability of AI/machine learning (AI/ML) in the medical field for the first time. They widened systematic medical image analysis by AI for computer-guided diagnosis and physician labor reduction [2]. Over time, AI has remarkably evolved from theory to practicing machine learning, expert systems, machine logic, neural networks, and the paradigm shift from “Rule-Based Systems” to “Data-Driven approaches” enabled supervised learning AI [3,4,5,6]. Notable global investments have been in advancing this disruptive technology [7]. The term “AI” is a diverse set of inquiries and development from various disciplines and conceptual strategies with no universally accepted definition, often seen as research that creates technologies capable of tasks requiring human-like intelligence [8]. Several cognitive scientists, AI experts, and philosophers suggest that AI research can provide insights into the workings of the human mind [9,10,11]. Some, like Turing, argue that if an AI behaves indistinguishably from a human, its intelligence should be considered natural [8,12]. Other key AI pioneers suggested alternative terms and definitions, such as “A thinking machine” [13] and “A general problem solver” [14].

AI is increasingly impacting all sectors of the economy, fueled by swift advancements in information processing and rising consumer expectations for competitive products and services. As AI is transforming the global economy, AI definitions have evolved to balance technical precision and accessibility. The Organization for Economic Co-operation and Development (OECD) [15] defines AI as ‘systems that can be regulated, certified, and put on the market, with a key focus on their disruptive economic potential’. The EU’s High-Level Expert Group on AI builds on this by highlighting the ability of AI to learn and adapt based on outcomes. The EU defines AI as ‘A system designed to operate autonomously, potentially showing adaptiveness post-deployment, by using machine or human-provided data to infer how to meet human-defined objectives through machine learning and logic approaches’ [16]. A more user-friendly definition offered by UNICEF describes AI as ‘machine-based systems that make predictions, recommendations, or decisions to influence environments, often appearing autonomous while still relying on human-defined objectives [17]. This definition is very close to the U.S. Food and Drug Administration’s (FDA) definition of AI [18]. This broader view accommodates data-driven, symbolic, and future AI paradigms, emphasizing the critical role of human oversight at every stage of AI-enabled development. As AI regulation progresses, debates over legal definitions and scope continue, reflecting the diverse techniques and technologies that comprise AI systems today. Regulatory agencies have issued papers addressing challenges in AI-driven therapeutic product manufacturing [19,20].

AI applications have been expanded into various domains and interdisciplinary fields, from computer sciences, languages, and statistical modeling to biology and healthcare, education, the pharmaceuticals industry, drug discovery and development, sales and marketing, business decision-making, finance, and beyond [19,20,21,22,23]. AI’s application in pharmaceuticals began in earnest in the late 1990s when advancements in machine learning and deep learning showed promise in drug discovery. Initially, AI was used primarily for data analysis and identifying potential drug targets. However, as algorithms became more sophisticated, AI’s role expanded to include compound screening, molecular design, and clinical trial design [24,25,26]. Pfizer and AstraZeneca are harnessing AI to accelerate drug discovery and improve patient outcomes. In collaboration with CytoReason, Pfizer is building a simulated immune system model to uncover new medicines and match treatments to patients more efficiently [27]. Similarly, AstraZeneca’s partnership with BenevolentAI focuses on using AI to identify drug targets in immunology and cardiovascular research [28]. Both collaborations highlight the need for AI-human partnerships, combining biology and data science expertise to reduce costs, speed development, and create transformative therapies.

Although AI holds great promise for advancing drug and biological product development, avoiding overreliance on these technologies in this highly regulated field is essential. While the health authorities’ approach supports AI innovation, AI systems are not infallible. They can make mistakes, and challenges persist, including the need for representative datasets, concerns about bias, and issues with interpretability [5,19,29]. Human oversight remains essential to ensure that AI-driven decisions are accurate and appropriate. The shift from rule-based to data-driven methodologies has profoundly transformed the AI landscape, unlocking new potential and steering ongoing research and development [16].

A well-structured governance framework for generative AI is critical for ethical and responsible technology use. Such a framework should include clear internal standards and guidelines prioritizing accountability, transparency, and regulatory compliance. By adopting best practices, organizations can establish a robust governance structure that supports responsible AI development and deployment. There is an ongoing need to ensure that the potential risks associated with AI are assessed and mitigated. Therefore, the FDA and EMA guidelines could benefit from greater specificity and clarity on potential biases or errors in data collection and analysis, providing more practical frameworks for careful monitoring and validation [16,19,29].

The US FDA has been proactive in establishing regulatory frameworks to guide the application of AI in healthcare and the pharmaceutical industry. To address emerging challenges, the FDA’s Center for Drug Evaluation and Research (CDER) launched the Center for Clinical Trial Innovation (C3TI) to foster innovative approaches in clinical trials and precision medicine. Despite these advancements, the FDA has faced criticism for delays in updating clinical trial guidance, highlighting the need for more robust and timely regulation in this rapidly evolving cyberspace [30].

Privacy is a particularly pressing concern. AI algorithms often rely on large datasets that may include sensitive patient information. This data could be compromised without proper safeguards, leading to significant privacy breaches. Ensuring data security and anonymization is crucial for protecting patient confidentiality. Furthermore, AI algorithms are only as good as the data on which they are trained. If the training data are biased, the AI system may perpetuate these biases, leading to unfair or discriminatory outcomes. To mitigate this risk, it is essential to use diverse and representative datasets and to regularly audit AI systems for bias [31]. The use of AI in healthcare also raises critical ethical questions. For instance, should AI be used to make life-or-death decisions? How can we ensure that AI systems are deployed relatively and equitably? Addressing these ethical concerns requires thoughtful consideration and collaboration among scientists, policymakers, and ethicists.

Navigating AI and ML technologies’ complex ethical and regulatory landscape in good manufacturing practices (GMP) and clinical practices (GCP) is indispensable for drug and biologics development organizations. Staying informed about evolving regulations, such as the EU’s AI Act and the US’s sector-based approach, is pivotal. Providing education and training to healthcare professionals and the public about AI’s benefits and risks is equally important. Ethical principles like fairness, transparency, and accountability are essential for responsible AI development. In August 2024, the FDA hosted a workshop on AI in drug and biological product development, co-organized with the Clinical Trials Transformation Initiative (CTTI). A group of regulatory and industry experts convened to underscore AI’s transformative potential in streamlining clinical trials, optimizing drug discovery, and improving patient outcomes [30]. Key takeaways included the importance of transparency, data quality, management, and algorithmic fairness, though the workshop did not fully address ethical implications related to bias and privacy protection. Integrating AI across diverse clinical settings and the need for multidisciplinary collaboration also require further attention to practical instruction on implementation [32,33].

Here, we employ a comparative lens to overview the current regulatory landscape and a synopsis of the FDA workshop discussions on the use of AI in drug and biological product development. We discuss the promises, challenges, limitations, related practices adopted to overcome them, and our practical recommendation for regulatory oversight. Finally, the paper outlines a path forward and future opportunities.

## 2. A Review of the FDA and CTTI Joint Workshop 2024: Keynote Speaker

On 6 August 2024, the FDA, in collaboration with the Clinical Trials Transformation Initiative (CTTI), hosted a hybrid workshop titled “AI in Drug & Biological Product Development”. This event focused on the responsible application of AI in drug development and manufacturing, discussing emerging AI technologies, associated challenges, and future directions. Experts from diverse fields contributed to the discussions.

The workshop opened with a keynote address that spanned the entire drug development lifecycle, covering early discovery, non-clinical studies, clinical research, late-stage manufacturing, and pharmacovigilance. The role of AI/ML technologies in enhancing these processes was highlighted. Emphasis was placed on the collaborative efforts between various FDA centers, including the Center for Drug Evaluation and Research (CDER), the Center for Biologics Evaluation and Research (CBER), the Center for Devices and Radiological Health (CDRH), and the Office of Product Quality (OPQ). These efforts focused on four key areas: (1) fostering collaboration to protect public health, (2) advancing regulatory approaches to support innovation, (3) promoting harmonized best practices, and (4) developing tools to evaluate and monitor AI performance.

**Figure 1:**
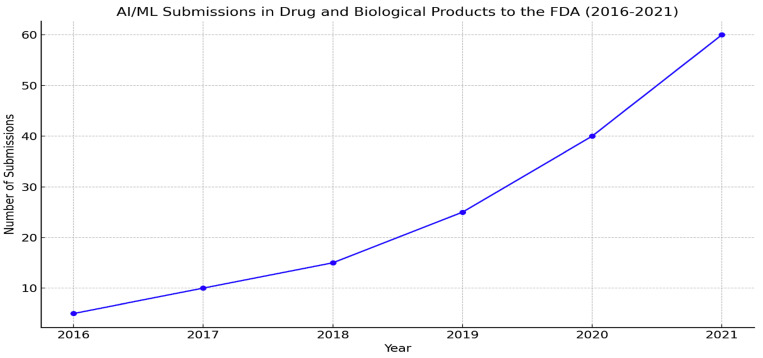
The increasing number of AI/ML submissions in drug and biological products to the FDA CDER from 2016 to 2021. The trend shows a significant rise in submissions over this period (adapted from Liu et al., 2022 [34]).

The FDA has already approved numerous AI/ML-based drugs and biological products. With the increasing number of clinical trials and high success rates, this number is expected to rise significantly [34]. In 2021 alone, over 100 FDA applications incorporated aspects of AI, highlighting its increasing role in drug and biological product development (Figure 1). The workshop underscored the importance of continuing risk-based regulatory approaches and engaging all stakeholders—academia, biotech, pharmaceutical companies, and international regulators—to advance regulatory science and support innovation in AI-driven drug development [35].

**Figure 2:**
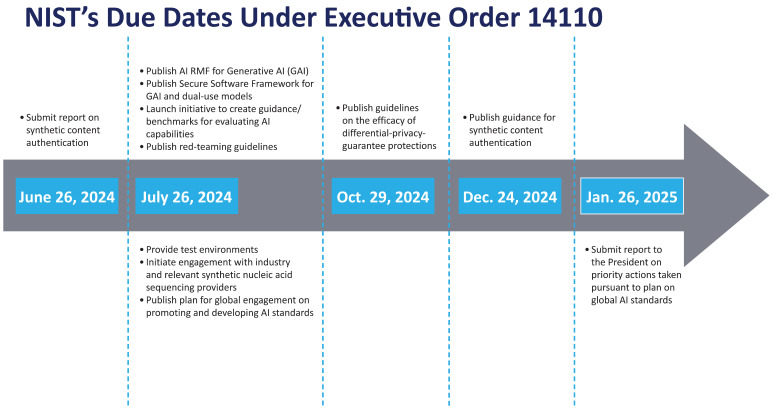
NIST due dates under Executive Order “14110” (adapted from the NIST website).

Integrating AI into drug development, manufacturing, and clinical trials is a focus for several FDA offices, including the CDER and CBER, as well as the National Institute of Standards and Technology (NIST). These agencies are advancing AI frameworks, guidelines, and standards to ensure the safe and effective use of AI in these areas (Executive Orders [36] (Figure 2).

The CDER has established a framework emphasizing the rigorous validation, data integrity, and transparency of AI systems. This framework integrates AI with continuous manufacturing and process analytical technology (PAT) to enhance process control and product quality. CDER also develops clear regulatory pathways for validation, monitoring, and post-market surveillance for AI-based systems. CBER has issued guidelines for AI in biologics manufacturing, prioritizing risk management to ensure product safety and efficacy. CBER supports continuous AI improvement in manufacturing, mainly through real-time monitoring and feedback mechanisms that enhance product quality. NIST is crucial in developing AI standards, focusing on data quality, algorithm performance, and transparency. NIST’s initiatives include developing performance metrics and best practices for validating AI systems to meet regulatory and scientific standards. These initiatives underscore a unified approach to AI regulation and implementation, emphasizing safety, ethical use, and international co-operation in the evolving landscape of drug development, manufacturing, and clinical trials (Table 1).

Additionally, the Executive Order on AI, issued in December 2023, provides a comprehensive government policy to address various AI-related legal and regulatory implications for the safe, secure, and trustworthy development of AI technologies in the healthcare industry, including drug and device development, personalized medicine, clinical trials, and patient monitoring. The Order emphasizes safety, security, ethical considerations, and the need for fairness and accountability in AI systems. It calls for increased AI research and development, regulatory frameworks, international collaboration, and public engagement to balance innovation with public safety and ethical norms. Ongoing monitoring and evaluation of AI systems are also highlighted to ensure their effectiveness and address emerging risks. It aims to balance innovation with public safety and ethical considerations [36]. Key points include:

- Safety and Security: The Order mandates the development of standards and guidelines to ensure safe and secure AI systems. This includes protecting against misuse, safeguarding data privacy, and ensuring AI operates transparently and reliably.
- Ethical Considerations: It emphasizes fairness, accountability, and transparency in AI systems to prevent discrimination and ensure responsible use.
- Research and Development: The Order calls for increased investment in AI research and development, encouraging collaboration among government, industry, and academia to advance technology while addressing safety and ethical concerns.
- Regulatory Framework for health care and drug development: This encompasses a wide range of activities, from clinical research to post-market surveillance, all subject to established regulations and FDA guidance. Relevant agencies are tasked with developing regulations and guidelines for AI development, deployment, and monitoring to ensure compliance with safety and ethical standards.
- Quality Assurance: The Department of Health and Human Services (HHS) is responsible for establishing a strategy to maintain quality in AI-enabled healthcare technology through premarket assessment and post-market oversight. The testing and validation standards required to ensure data quality, reliability, reproducibility, and accuracy across drug development are also crucial. Incorporating open-source and real-world data (RWD) into AI model development and appropriate documentation related to data source selection, inclusion, and exclusion is essential for effective AI implementation. Understanding how quality standards will impact overall product development requirements, both within and outside the U.S., is vital. Furthermore, determining the necessary transparency and reporting requirements to address trends and propose changes considering post market safety issues or other RWD is crucial.
- International Collaboration: The Order promotes international co-operation to establish global standards and best practices for AI.
- Public Engagement: It supports engaging the public and stakeholders in discussions about AI technologies, promoting transparency, and involving various communities in decision-making.
- Monitoring and Evaluation: Ongoing monitoring and evaluation of AI systems are outlined to assess their impact and effectiveness, with policies and regulations adapting based on emerging technologies and societal implications.

Overall, the Executive Order seeks to foster innovation in AI while addressing potential risks and ensuring that AI technologies are developed and used responsibly for societal benefit. Incorporating these elements, the combined perspectives highlight a unified approach to AI regulation and implementation across various agencies, emphasizing safety, ethical use, and international collaboration.

## 3. The Broader Role and Applications of AI in Drug and Biologics Development: Lessons from the FDA Workshop and Industry

The pharmaceutical industry has long relied on trial-and-error methods for drug discovery, which, while sometimes effective, are often inefficient and costly. AI’s expanding portfolio of applications and methodologies is revolutionizing drug discovery and development, driving more efficient, precise, and cost-effective processes. Leading pharmaceutical companies have successfully integrated AI and ML across various stages of drug development, accelerating the identification of therapeutic targets, optimizing clinical trial design, and enhancing the drug pipeline. Below are critical real-world examples that highlight the impact of AI in drug discovery, followed by an exploration of the broader role AI plays in optimizing the drug development process. Moreover, AI’s ability to learn from existing data allows it to improve over time, making it an invaluable tool in the iterative drug development process. By continuously analyzing the outcomes of previous experiments, AI can refine its predictions, identifying more effective compounds with fewer side effects. This iterative learning process is a significant departure from traditional methods, where each new experiment often starts from scratch without the benefit of insights gained from previous failures [25].

Several key pharmaceutical industry players have demonstrated AI’s applications in drug and Biologics discovery. Bristol-Myers Squibb, for instance, deployed an ML model to predict CYP450 enzyme inhibition, which is essential for drug metabolism. Their AI tool achieved 95% accuracy, reducing failure rates by sixfold compared to traditional methods [37,38]. This improvement accelerated the screening process and allowed researchers to focus on drug candidates with a higher likelihood of success during human trials and FDA approval. Merck and Bayer partnered with Cyclica to enhance drug candidate identification through AI. This collaboration led to the discovery of a target protein linked to FDA-approved drugs for treating systemic scleroderma and the Ebola virus [39]. The use of AI to repurpose existing drugs highlights the technology’s potential to expedite the drug development cycle. In another example, GlaxoSmithKline (GSK) worked with Exscientia to discover a novel molecule targeting a pathway involved in chronic obstructive pulmonary disease (COPD). This collaboration demonstrated AI’s ability to identify innovative therapeutic targets for complex diseases [40].

Similarly, Exscientia and Evotec applied AI to discover a cancer treatment that targets the A2a receptor rapidly. Within just eight months, the drug candidate entered clinical trials in 2021, showcasing how AI can significantly reduce the discovery phase in drug development [41]. Berg’s AI platform identified BPM31510 as a promising treatment for advanced pancreatic cancer, one of the most challenging diseases. The platform’s capability to predict patient responses and potential adverse effects underscores AI’s growing role in enabling personalized medicine [42]. Rapid advancements in the use of AI in cancer research, diagnosis, and treatment have spanned a diverse range of indications. Numerous startups are leveraging AI to offer innovative approaches to transforming the field [43]. MultipleAI, for example, provides a comprehensive whole blood screening test that utilizes RNA sequencing technology and AI to detect a wide range of complex diseases, including cardiovascular and cancer [43]. Another notable achievement in AI-driven drug discovery came from BenevolentAI, which used its platform to identify baricitinib, initially developed by Eli Lilly for rheumatoid arthritis, as a potential treatment for COVID-19. The drug was subsequently approved for emergency use in the U.S. and Japan, illustrating how AI can rapidly repurpose existing drugs in response to global health crises [44]. Lastly, in collaboration with Insilico Medicine, Taisho Pharmaceutical utilized AI to identify compounds targeting cellular aging. Insilico’s AI system helped discover drug-like molecules that target senescent cells, which play a role in aging-related diseases [45]. This project underscores AI’s potential to pioneer new therapeutic areas, such as anti-aging therapeutics.

**Figure 3:**
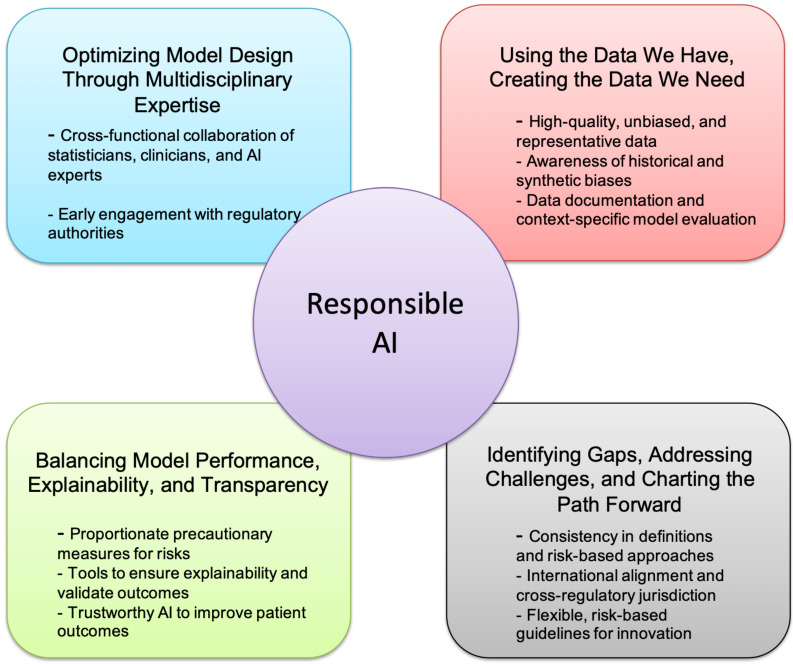
Key takeaways for the FDA workshop discussions on the use of “AI in Drug & Biological Product Development”.

This rapid integration of AI and ML technologies into drug development has the potential to revolutionize the pharmaceutical industry. However, adopting these advanced tools is fraught with complex technical, regulatory, clinical, and ethical challenges. The recent FDA workshop provided a forum for leading experts across academia, industry, and regulatory bodies to discuss these challenges and propose actionable pathways forward [30]. The workshop discussions focused on four major challenge areas that are critical to ensuring the safe, effective, and equitable deployment of AI/ML technologies in drug development (Figure 3 and Table 2):

### 3.1. Optimizing Model Design Through Multidisciplinary Expertise

A key theme of the workshop was the importance of integrating diverse expertise in designing AI/ML models. Developing AI solutions for drug development requires the collaboration of professionals from computational sciences, clinical research, regulatory affairs, and ethics to ensure that models are technically sound, clinically relevant, and compliant with regulatory standards. Several speakers emphasized that this multidisciplinary approach is vital to creating models that align with the realities of clinical practice and regulatory expectations [18,46] (Figure 3).

AI model optimization focuses on refining algorithms to improve performance, reduce computational costs, and ensure accuracy in real-world applications. Recent trends show a dramatic decrease in large language model sizes—nearly 90% between May 2022 and April 2023—signaling a shift toward greater efficiency and ease of deployment. Optimization strategies include hyperparameter tuning, feature engineering, transfer learning, and architecture refinement [47,48]. Key techniques involve retraining models with high-quality data, balancing model complexity and performance, and pruning non-essential features to enhance speed. Addressing overfitting through regularization and leveraging improved hardware configurations are critical to achieving optimal results. While traditional offline testing is instrumental in refining models, its limitations—such as time intensity, isolated metric focus, and lack of user feedback—highlight the need for online experimentation. Real-world user interactions provide invaluable insights, enabling developers to enhance usability and performance [48].

AI’s transformative potential extends to clinical trials. In clinical research, tools like Criteria2Query and TrialGPT exemplify how AI-driven systems enhance trial designs by automating patient matching, reducing reliance on control groups, and addressing biases to promote inclusivity [49,50]. These advancements, coupled with decentralized trials and remote monitoring, underscore the importance of multidisciplinary collaboration among scientists, clinicians, NIH, and regulators to align technical innovation with patient-centric outcomes [50].

The multidisciplinary approach remains critical for advancing AI in clinical trials and drug development. Collaboration among computational scientists, clinical researchers, regulatory experts, and ethicists ensures that AI/ML models are technically robust, clinically relevant, and aligned with regulatory standards. As decentralized trials, remote monitoring, and generative AI capabilities grow, these collaborative efforts will shape a future where innovation aligns seamlessly with clinical and regulatory realities [51].

### 3.2. Using the Data We Have, Creating the Data We Need: Clinical Development, Clinical Data Management, and Analysis

AI/ML technologies are poised to transform clinical trial design, patient recruitment, and data analysis. However, their success relies heavily on high-quality data, effective management, and mitigating biases that may compromise the accuracy and equity of AI-driven outcomes. Workshop participants highlighted the need for rigorous data governance practices, including inclusive data standardization, harmonization across regulatory frameworks, and fit-for-purpose approaches tailored to specific clinical applications [52]. AI bias refers to systematic patterns of errors in machine learning predictions, where models perform accurately in some scenarios but fail in others due to skewed or incomplete training data. These biases often align with specific data attributes, perpetuating social, cultural, and economic inequities. For example, an algorithm designed to detect breast cancer may reflect biases in the training data, leading to disparities in diagnostic accuracy across different demographic groups [53].

**Figure 4:**
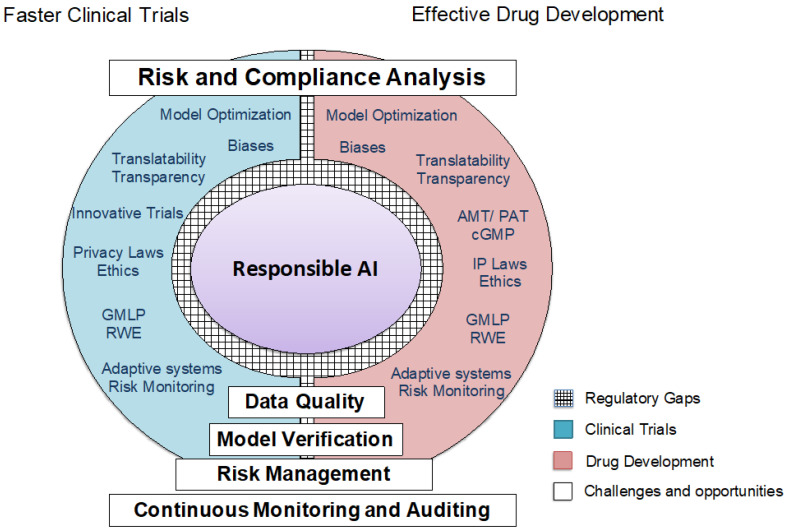
Future trends in AI regulations: establishing responsible data-driven AI for drug development. Good machine learning practices (GMLP) and AI-driven RWE usage.

The session addressed selection and algorithmic biases that can affect outcomes in clinical data analysis, and focused on ensuring clinical data integrity and model validation concerning FDA regulations on Good Clinical Practice [54]. AI aids in target identification, dose optimization, and understanding pharmacokinetics and pharmacodynamics [55]. Moreover, different datasets aggregate single-cell data from various research efforts, enabling in silico drug targeting and enhancing the precision of drug development and therapeutic targeting [56,57]. These predictions depend significantly on the volume and quality of the data used (Figure 3 and Figure 4, see Section 3.5).

In the discovery stage, AI facilitates target identification, dose optimization, and the analysis of pharmacokinetics and pharmacodynamics. Integrating large-scale datasets like those from the UK Biobank, FinGen, and PharmA enables predictive analyses like polygenic risk scores (PRS) for disease risk and advancing personalized medicine horizons. However, these predictions hinge on data quality and representativeness, requiring comprehensive coverage of relevant variables, disease states, and outcomes [30,58,59].

Clinical trial development represents another critical area where AI revolutionizes patient recruitment, trial design, and optimization through digital twins. Yet, biases such as demographic underrepresentation and site-specific disparities threaten to undermine AI applications in this phase. Workshop discussions emphasized the importance of rigorous data governance practices, including standardization, harmonization across regulatory frameworks, and fit-for-purpose approaches aligned with clinical objectives, FDA regulations on Good Clinical Practice (GCP), and newly revised ICH E6 GCP guidelines [60]. Addressing these biases demands an interdisciplinary approach to ensure equitable representation of subgroups in data and outcomes.

In manufacturing and post-market monitoring, AI improves quality control, pharmacovigilance, and real-world outcome analysis, enabling ongoing assurance of drug safety and efficacy [61]. Single-cell data aggregation from research efforts has also enhanced in silico drug targeting, aiding precision drug development. However, integrating such datasets requires vigilance to avoid biases introduced during data collection, curation, or merging incompatible sources. AI bias in advanced therapies manufacturing, such as cell and gene therapeutics, can have far-reaching consequences, from clinical trials to treatment outcomes. The development, manufacturing, and monitoring processes for these advanced medicines are more complex, with lengthy regulatory approval timelines facing substantial patient waiting time. These treatments are often costly, with some costing several hundred thousand dollars, making them unaffordable for public healthcare systems [61]. As a result, access is limited, creating a significant potential source of bias where only wealthier individuals can afford these medications. Moreover, the efficacy and safety of cell and gene therapies can vary across ethnic and genetic backgrounds; algorithms trained on non-diverse datasets risk overlooking these differences, further perpetuating health inequities. Addressing these biases is essential to ensure fairness, equity, and regulatory compliance in AI-driven therapeutics.

Bias is not a new challenge in the life sciences. Observer biases have long compromised data quality and contributed to the “reproducibility crisis”, where many scientific results cannot be replicated. Historical and synthetic biases compound the challenges in applying AI to drug development. Historical biases embedded in legacy data reflecting systemic disparities can perpetuate inequities in model predictions. For example, underrepresenting minority groups in datasets leads to less accurate predictions for these populations. Synthetic biases arise from data collection and processing errors, such as selection biases or inconsistent measurement tools, compromising generalizability and model reliability. Biases in AI also arise from assumptions embedded in algorithm design. Biased datasets may underrepresent specific populations, resulting in clinical trials lacking diversity and limiting therapies’ generalizability.

Similarly, biased AI systems used in healthcare resource allocation could exacerbate disparities by prioritizing certain patient groups over others. For instance, if developers assume specific symptoms are more prevalent in one demographic group, such as non-Hispanic White women, the algorithm may yield inaccurate predictions for others, like Black/African American women, or LGBTQ+ individuals. These biases reinforce systemic inequities, underscoring the importance of interdisciplinary approaches to model development and validation. Healthcare algorithms and AI can inadvertently exacerbate health disparities, especially for populations differentiated by race, ethnicity, gender, age, or other demographic factors. A primary cause is the lack of diversity in training data, which limits the algorithm’s ability to generalize across diverse patient groups. Ensuring equitable outcomes requires incorporating data from underrepresented populations and rigorously testing algorithms for demographic inclusivity [31,53].

Ultimately, integrating AI into drug development hinges on interdisciplinary collaboration, fostering data integrity, and achieving equity in outcomes. By recognizing and addressing biases, researchers and regulatory authorities can unlock the full potential of AI, advancing innovation while safeguarding public health (Figure 3 and Figure 4).

To address these issues from the technical and regulatory views, several strategies are critical to establishing a data-driven, responsible AI (Figure 4):

- Mitigating historical biases involves augmenting datasets to include underrepresented populations and ensuring fair representation of diverse demographic groups.
- Awareness of synthetic biases requires robust documentation, transparent reporting, and harmonized data standards to ensure the compatibility and reliability of combined datasets.
- Data-driven validation practices must align with regulatory standards, employing algorithm explainability and trustworthiness verification to maintain confidence in AI outputs.

While biases like confirmation, hindsight, and anchoring are inherent in human decision-making, AI systems offer the advantage of detailed scrutiny. Algorithms can be refined by identifying and addressing these biases to ensure more objective and equitable outcomes. Regulatory initiatives are increasingly focused on addressing bias in AI systems. The STANDING Together initiative, launched in 2022, aims to set standards for data diversity, inclusivity, and generalizability in medical AI by recommending guidelines for dataset composition and reporting [62]. The FDA has also prioritized this issue through its Action Plans, emphasizing bias detection and mitigation. Key measures include piloting real-world performance monitoring and introducing draft guidance to streamline regulatory processes for iterative AI model improvements while ensuring safety and effectiveness [18]. These efforts are crucial to minimizing bias without stifling AI innovation (Figure 4).

### 3.3. Balancing Model Performance, Explainability, and Transparency

The trade-off between high model performance and the need for transparency and explainability emerged as a central topic of discussion. While AI models, particularly those based on deep learning, can achieve high levels of accuracy, they often operate as “black boxes”, limiting their interpretability. Speakers emphasized that explainability is critical for regulatory approval and clinical adoption, as stakeholders must be able to trust and understand the outputs of AI models. The workshop underscored the need for innovative solutions, such as dual-model approaches and transparency tools, to balance these competing demands [63,64,65].

Explainable AI (XAI) refers to the ability to describe an AI model, its potential biases, and its expected impact. It ensures transparency, fairness, and accuracy in AI-driven decision-making, fostering trust and confidence when deploying AI models. By promoting clarity, XAI supports organizations adopting a responsible and ethical approach to AI development. Therefore, XAI addresses technological challenges and raises critical medical, legal, ethical, and societal questions requiring thoughtful consideration. It is essential to align XAI with trustworthiness requirements, particularly in high-risk scenarios such as healthcare. AI/ML model validation is critical for ensuring reliability and trustworthiness. Evaluating performance on unseen data reveals the model’s strengths, limitations, and potential issues. This process supports informed decisions about deployment, ensuring the model’s effectiveness in practical applications. A detailed case study on AI trustworthiness in healthcare highlights the importance of regulatory compliance, as outlined in frameworks like the EU AI Act [16]. By ensuring transparency and accountability, XAI fosters responsible AI development and reinforces trust in its applications (Figure 4).

As AI evolves, balancing efficiency with transparency ensures its potential is fully realized in transforming healthcare and beyond. AI significantly reduces the manual effort required to optimize and implement eligibility criteria for clinical trials, swiftly identifying eligible patients once criteria are established (Figure 3).

Beyond efficiency, AI/ML applications increasingly demand XAI to ensure transparency, trustworthiness, and accountability. XAI achieves this by embedding prior knowledge into interpretable, data-driven algorithms, flagging untrustworthy inferences, and employing techniques such as “learn to optimize” (L2O) for solving optimization problems. Trust is further reinforced through interpretable certificates, which validate model predictions, with applications spanning clinical trial matching, medical imaging, and even complex domains like arbitrage trading of crypto assets [51,66].

XAI is central to implementing responsible AI and ensuring fairness, accountability, and transparency in large-scale applications. Responsible AI frameworks encourage organizations to embed ethical principles into AI processes, fostering trust and transparency (Figure 3 and Figure 4). In healthcare, XAI enhances model interpretability while maintaining accuracy, enabling clinicians to understand AI-driven insights better. Advanced AI techniques can improve predictive accuracy and aid in early diagnosis and personalized treatments. Involving subject matter experts in feature selection and presenting outputs in clinician-friendly formats, such as forest plots, can foster trust and informed decision-making. Transparency and ethical practices are essential to addressing biases and concerns of fairness and building confidence in AI systems [30].

### 3.4. Identifying Gaps, Addressing Challenges, and Charting the Path Forward

Despite the progress made in AI/ML regulatory frameworks, significant gaps remain, particularly in the harmonization of global policies and the continuous validation of AI models post-market. The workshop discussions called for a coordinated, international effort to develop more consistent and fit-for-purpose pathways for AI applications in drug development. Regular model monitoring, the integration of real-world data, and clear guidelines for risk-based approaches were identified as critical components for advancing AI’s potential while safeguarding patient safety.

Through these four challenge areas, the FDA workshop provided a comprehensive overview of the current state of AI/ML in drug development and identified key priorities for future research, policy development, and cross-sector collaboration (Figure 3 and Table 2). By addressing these challenges, the AI community can work toward a future where AI/ML technologies contribute meaningfully to faster, safer, and more personalized drug development. We will discuss some of these challenges in the following sections.

### 3.5. Data Integrity and Quality Challenges in AI-Driven Drug Development Governance Considerations: Practical Guidelines for AI Implementation

Data integrity and quality are critical factors in the success of AI-driven drug development. Data accuracy, consistency, and completeness are essential for training effective AI models and generating reliable results [52]. Data integrity and quality challenges can arise from various sources, including data collection errors, biases, and inconsistencies across different datasets [67,68,69,70,71]. Addressing the following data-related challenges is crucial for developing AI models that can provide meaningful insights and support informed decision-making throughout the drug development process:

One of the primary data-related challenges in leveraging AI for drug development is the absence of standardized sources for patient demographic data. This lack of standardization complicates efforts to set accurate enrollment goals for underrepresented groups in clinical trials, a crucial aspect of ensuring that trials are diverse and equitable. For AI applications, standardized data are essential for training models representative of the target populations. However, disparate and often incomplete data sources significantly hinder the integration and analysis processes required for effective AI-driven drug development. The resulting gaps in data availability can lead to models that fail to generalize across diverse groups, limiting their potential to drive genuinely inclusive innovations.

A related challenge is the insufficient biomarker data across demographic groups, which further hampers the development of AI systems capable of predicting drug efficacy and safety for diverse populations. AI models rely on extensive and diverse biomarker data to accurately generalize their predictions across various population subsets. The current gap in biomarker data affects clinical endpoint assessments and impedes the generation of accurate individual case safety reports (ICSR). This lack of comprehensive biomarker data hampers the ability of AI systems to predict drug efficacy and safety for diverse populations accurately. AI models require extensive and diverse biomarker data to generalize across various groups and provide accurate insights on the adverse effects of drugs, as well as automated case submission and evaluation.

Another significant obstacle is the inconsistency in the definitions of race and ethnicity across different datasets. This lack of uniformity complicates data integration and analysis, which is particularly problematic for AI applications in drug development. Consistent and clear definitions of race and ethnicity are critical for ensuring that AI algorithms are trained on relevant and comparable data, which helps to avoid perpetuating existing biases. Inconsistent definitions increase the risk of reinforcing these biases in AI models, potentially leading to skewed outcomes that fail to address the needs of diverse populations. Moreover, the inadequate collection of social determinants of health (SDoH) data further limits the ability of AI models to account for the vast array of factors that influence drug responses and overall health outcomes. Social determinants of health, such as socioeconomic status, education, and access to healthcare, play a critical role in determining patient outcomes. Incorporating SDoH into AI models is essential for understanding the broader context of patient health and ensuring that drug development efforts are inclusive and effective across diverse populations. Unfortunately, these data are not routinely collected, presenting a substantial barrier to developing AI systems that reflect real-world complexities.

Additionally, the limited availability of robust data on populations outside the United States poses a significant challenge to the global applicability of AI models in drug development. For AI systems to drive innovation worldwide, they must be trained on diverse datasets that include international populations. Without such data, the findings produced by AI models risk being overly specific to U.S. healthcare systems. They may fail to generalize to other regions, limiting the broader utility of these models in international drug development efforts. Addressing these data challenges is critical for the effective implementation of AI in drug development. One proposed strategy involves creating a centralized repository for biomarker data, which would serve as a standardized source of nationally representative data for various disease areas. This repository could integrate race and ethnicity data, enhancing AI models’ ability to generate inclusive and accurate insights. By consolidating existing data sources and developing integration standards, researchers can overcome inconsistencies and support the development of more robust AI models. This would provide a more comprehensive understanding of disease biology and facilitate the identification of promising drug targets.

Beyond these strategies, additional challenges must be addressed to optimize AI use in drug development. Data privacy and security are paramount, particularly given the sensitive nature of patient data used in training AI models. Ensuring compliance with data protection regulations requires the implementation of robust encryption, anonymization techniques, and transparent data governance policies. Furthermore, navigating the evolving regulatory landscape for AI applications remains a significant challenge. Proactive engagement with regulatory agencies and participation in industry forums are necessary to stay abreast of changes and ensure that AI innovations are aligned with regulatory expectations. Integrating AI tools into existing drug development processes and scaling them across different markets presents further obstacles. For AI to reach its full potential, solutions must be scalable and adaptable to various production environments, including diverse regulatory and healthcare systems. The FDA’s discussion paper on AI and ML in drug development underscores the agency’s commitment to a risk-based regulatory framework that fosters innovation while prioritizing patient safety. This framework spans the entire drug development lifecycle, covering drug discovery, non-clinical research, clinical research, post-market safety surveillance, and advanced manufacturing technologies (AMT).

Several key considerations are vital for successfully implementing AI/ML in drug development. First, AI systems must be developed and deployed with human-led governance, accountability, and transparency to ensure responsible and ethical use. Additionally, the quality, reliability, and representativeness of data used in AI models must be guaranteed to ensure that the models reflect the diverse populations they aim to serve. Lastly, rigorous model development, performance monitoring, and validation processes ensure that AI systems deliver accurate, reliable, and actionable insights throughout drug development [46,47,48,49,50].

The successful integration of AI in these areas requires adherence to best practices in data management, model validation, transparency, risk assessment, management approaches, continuous monitoring, and strategic collaboration. By addressing these fundamental considerations, organizations can harness AI’s potential to accelerate drug discovery, improve patient outcomes, and drive innovation in the healthcare industry. As an example of successful AI tools in drug development, Cassandra- an ICON’s advanced AI prediction system- demonstrates how integrating real-world evidence with regulatory insights can streamline postmarking requirements, enhance regulatory approval, and facilitate a more efficient path to market [72].

### 3.6. Access, Fairness, and Accountability: Lessons from Economic, Law, Ethics, and Politics

Integrating AI into drug development and manufacturing offers transformative economic potential, reducing the cost and time to market for new therapies. AI algorithms can optimize drug formulation and predict clinical trial outcomes, cutting research and development costs. In manufacturing, AI-driven platforms enhance efficiency, improve quality control, and support real-time monitoring. These platforms, recognized by the FDA’s advanced manufacturing platform designation [73], enable cost-effective, flexible production even for complex biologics [25,74]. However, AI/ML systems and algorithms used in drug development and manufacturing can resemble the economic profile of a “natural monopoly”, akin to railroads or telecommunications [75,76,77]. The high fixed costs of AI development, low marginal deployment costs, and network effects allow companies controlling these platforms to dominate the market [77,78,79]. This economic structure poses regulatory challenges, requiring thoughtful oversight to prevent monopolistic pricing and ensure access [78,80,81,82].

AI/ML can be thought of as two distinct workflows: first, the design, development, and training of a model, and second, the deployment of that model to make decisions in real-world scenarios [82]. For companies, there are no disadvantages to collecting unlimited data; in the United States, few restrictions prevent them from doing so. As AI becomes central to biopharmaceutical production, regulatory oversight, not competition, may be needed to maintain fair pricing and equitable access to balance monopolism in this data-intensive industry [76]. Drawing on historical lessons from other monopolies, regulation can prevent practices like inflated pricing or restricted access. Moreover, it can address issues like data privacy, algorithmic bias, and prioritizing profit over patient care [79,83,84]. We will discuss HIPAA and GDRP as examples of such a provision. Public oversight will ensure that AI’s benefits are distributed across the healthcare ecosystem, not concentrated in a few dominant entities [85].

One primary concern with natural monopolies, including AI, is the potential for inefficiently high pricing. AI-driven drug development could result in exorbitant fees for access to platforms or data. Regulators can mitigate this by implementing rate regulation—similar to traditional approaches with natural monopolists—to protect consumers and limit data collection to what’s necessary for system improvement. This would address privacy concerns and prevent developers from extracting personal information in exchange for services, which could deter privacy-conscious users from benefiting from AI [84]. Another challenge is the risk of underinvestment in areas like safety, security, and bias prevention. Monopolists in concentrated markets have less incentive to improve safety or address discrimination, which is especially dangerous in healthcare. Regulators could impose service-quality standards, requiring AI systems in drug development to meet accuracy, reliability, and fairness benchmarks, ensuring AI aligns with social goals like accountability and equity [86]. AI monopolies can also lead to inefficiencies in competition, mainly through wasteful data collection and duplication of infrastructure investments. To promote competition without this waste, regulators could adopt franchise bidding or mandate data-sharing and federated learning, allowing multiple competitors to access shared datasets and infrastructure.

While AI has the potential to revolutionize drug development and lower therapeutic costs, its monopolistic tendencies demand robust regulation. Policymakers can ensure AI-driven innovations serve the public good without harming consumer welfare by addressing concerns over data privacy, underinvestment, and inefficient competition. AI technologies should primarily operate under a policy framework that encourages permission-less innovation while counseling against “command-and-control regulation”, enabling humanity to fully harness the opportunities and benefits they offer [87,88]. With appropriate oversight, the benefits of AI in FDA-recognized manufacturing platforms can be realized while preventing the downsides of unchecked monopolies.

### 3.7. Navigating the Future—GDPR Compliance and Harmonization in Clinical Trials

The regulatory landscape for generative AI is rapidly evolving, with different countries and regions adopting varying approaches. In the United States, the FDA has issued guidance on using AI in medical device development [18], emphasizing the need for transparency, validation, and clinical evidence. The European Union has proposed the AI Act [16], establishing a risk-based framework for regulating AI systems. The landscape of clinical trials is undergoing a profound transformation with the integration of AI. This technology promises to enhance drug development processes but also introduces complex challenges related to data protection, regulatory compliance, and harmonization across jurisdictions.

Several emerging regulations and policy initiatives are shaping the future of generative AI in drug and biologics development [30]. These include addressing biases in AI algorithms to ensure equitable outcomes, protecting intellectual property rights related to AI-generated content, ensuring AI systems can provide understandable explanations for their decisions, and fostering collaboration between regulators, industry, and academia to develop effective governance and regulatory frameworks.

### 3.8. Ethical and Compliance Challenges on AI’s Expanding Role in Clinical Trials

The ethics of AI remains one of the critical implications in AI-driven biopharma. Notably, in more complex areas such as cell and gene therapy, analytics based on probabilities often clash with traditional deterministic approaches, creating challenges for adoption [61,74]. Unaddressed biases in AI can exacerbate disparities in disease diagnosis or drug recommendations. It is essential to establish clear guidelines on when and how patients are informed about the role of AI in their treatment, clinical trials, or diagnostics to ensure transparency and trust. AI is increasingly used to optimize various aspects of clinical trials, from study design to patient recruitment. By analyzing large datasets, AI can help researchers design more effective trials, predict outcomes, and refine protocols [53]. In inpatient recruitment, AI’s ability to process electronic health records and other data sources streamlines the identification of eligible participants.

Additionally, AI-powered digital health technologies (DHTs) facilitate continuous monitoring, making decentralized trials more feasible and inclusive. Despite its potential, the integration of AI raises significant ethical and global regulatory concerns, particularly around disparities in clinical studies [89], data privacy, and security [32]. Ensuring compliance with existing regulations is crucial to protect patient information and maintain trust in the research process.

### 3.9. Ethical and Legal Considerations of Privacy and Nondiscrimination

Fundamental ethical principles, including transparency, fairness, and accountability, must govern AI’s application in clinical trials. Compliance with regulations such as the General Data Protection Regulation (GDPR) and the Health Insurance Portability and Accountability Act (HIPAA) is essential for protecting patient privacy [90,91]. GDPR, with its rigorous data protection standards, influences global AI governance and sets a benchmark for data privacy. Data privacy and security are paramount in clinical research. The collection and use of personal health information require strict adherence to regulations to mitigate legal risks and maintain participant trust [92]. The executive order prioritizes individual privacy and non-discrimination in AI-enabled technology. It calls for comprehensive privacy legislation and emphasizes the need to mitigate biases and prevent discrimination in AI applications.

Patients who are not informed about potential biases in AI models may be unable to provide fully informed consent. At the same time, inaccurate treatment recommendations could breach HIPAA regulations or lead to FDA recalls. Additionally, biased AI systems can expose healthcare providers and developers to legal liabilities, including medical malpractice claims, if harm occurs due to biased predictions. Notably, in cell and gene therapy, the limited availability of training data—often restricted to small patient populations—exacerbates these challenges, leading to flawed assumptions about immunogenicity, efficacy, and patient selection. For instance, prostate cancer treatment models trained on homogeneous datasets may fail to account for ethnic, cultural, or dietary differences, resulting in inaccurate predictions when applied to more diverse populations. Mitigating bias often involves complex trade-offs, such as balancing models tailored to specific subgroups, which may reduce statistical power, or enhancing general models with additional variables at the risk of overfitting.

Addressing these biases requires diverse, comprehensive datasets and continuous testing. While achieving perfectly unbiased models may remain unattainable, identifying and understanding biases is a crucial first step. By integrating AI insights with expert medical judgment and refining methodologies for representational fairness, monitoring, and auditing AI-driven pipelines, as well as more trustworthy AI, we can minimize disparities and improve outcomes in healthcare [61,89].

## 4. Regulatory and Compliance Framework for AI in Drug Development

The regulatory environment for using AI in drug development and clinical trials rapidly evolves to address these technologies’ unique challenges and opportunities. In the European Union, the EU Artificial Intelligence Act classifies AI systems according to their risk levels and sets stringent requirements for high-risk applications [16]. This framework emphasizes vital principles such as transparency, accountability, and data protection, which are essential for the ethical application of AI in healthcare settings.

The FDA’s discussion paper on AI and ML applications in drug development and clinical trials in the United States outlines a comprehensive approach to regulating these technologies. The FDA defines AI as a broad category encompassing algorithms and models capable of learning, decision-making, and prediction [19]. ML, a subset of AI, creates models through data analysis. The FDA’s discussion paper articulates a commitment to a risk-based regulatory framework that supports innovation while prioritizing patient safety. It covers a wide array of drug development activities—from initial discovery through post-market surveillance—and explores how AI/ML can be integrated across various stages, including drug discovery, non-clinical and clinical research, post-marketing safety monitoring, and advanced manufacturing. While recognizing the potential benefits of AI and ML, the FDA also addresses the risks associated with these technologies, such as data biases and limited explainability of models. The discussion paper calls for clear guidance and standards for the diverse applications of AI throughout the drug development process. It identifies critical concerns such as data quality, model reliability, privacy, and transparency and emphasizes the need for stakeholder engagement to address these issues. The FDA’s approach includes fostering dialogue with industry, academia, and other stakeholders to develop guidelines that ensure AI’s ethical and practical use in drug development [19,20,30].

The discussion paper also highlights the transformative potential of AI and ML to enhance various aspects of clinical trials, including participant selection, data management, and trial design [30]. AI technologies can optimize participant selection by analyzing vast datasets to identify suitable candidates, thereby improving the diversity and representativeness of trial populations [31]. This is crucial for addressing historical bottlenecks in patient recruitment and retention, which have often slowed the completion of clinical trials. Additionally, AI/ML can support innovative trial formats, such as decentralized clinical trials (DCTs), which utilize digital health technologies (DHTs) to facilitate more participant-centric approaches. These non-traditional trial designs can increase accessibility and reduce participant burden, leading to more efficient and inclusive trials [30].

The FDA acknowledges the potential of AI/ML to improve trial design efficiency by using RWD and other data sources to predict effective study protocols and identify optimal participants. However, the FDA also emphasizes the need to maintain high standards for data quality and ensure robust oversight to mitigate bias and data reliability risks. The agency stresses the importance of ethical considerations, privacy protections, and transparency in developing and using AI technologies in clinical trials [30,51,92]. AI technologies have the potential to revolutionize clinical trials by extracting valuable data from unstructured reports and automatically annotating images or lab results. AI can fill in missing data points through predictive modeling, allowing researchers to identify unique subgroups within a population that respond differently to treatments.

Additionally, AI techniques can extract critical information from clinical trial reports, including recovery outcomes, symptoms, side effects, and adverse incidents. This data extraction process can streamline the formatting of eligibility requirements from trial descriptions into structured tables, optimize site selection, refine eligibility criteria, and predict trial outcomes. Tasks traditionally requiring a team of data scientists, such as data analysis or visualization coding, can now be automated with AI, significantly enhancing efficiency and reducing costs [93].

Integrating AI into healthcare brings significant benefits but poses serious privacy concerns. The rise of large language models further expands the possibilities for AI in clinical trials. These models can support healthcare professionals in various capacities, such as early disease detection, medical image interpretation, drug discovery, treatment recommendations, and remote patient monitoring. By automating repetitive tasks and providing decision support, AI can enable more accurate and timely diagnoses, ultimately improving patient care and optimizing healthcare resource allocation. This technological advancement facilitates a more personalized approach to medicine, where treatments can be tailored to the specific needs of patients, enhancing both efficacy and safety. As commercial entities gain increased access to patient health data, there is a growing risk of misuse or insufficient protection of this sensitive information. The “black box” nature of many AI algorithms complicates oversight, as their decision-making processes can be opaque [91]. Regulations must emphasize patient agency to address these challenges, including recurrent informed consent and the right to withdraw data. Additionally, innovative data protection measures and strict jurisdictional controls are needed to safeguard privacy in an evolving technological landscape. Without robust oversight, we risk falling behind the rapid pace of AI development.

The FDA has approved AI/ML applications in clinical care, such as software to detect diabetic retinopathy from diagnostic images. This approval marks a significant step towards integrating AI into everyday medical practices. Despite the promising capabilities of AI in healthcare, many technological advancements still originate in academic research settings. Partnerships with commercial entities are often necessary to bring these innovations from the lab to real-world application. These collaborations can help scale AI technologies and ensure they are accessible and beneficial to the broader healthcare community [94,95].

The European Medicines Agency (EMA) has also been proactive in this area, issuing a draft reflection paper on the application of AI and ML in developing, regulating, and using human and veterinary medicines. This document, part of the Heads of Medicines Agencies (HMA)-EMA joint Big Data Steering Group’s Workplan 2022–2025, explores the role of AI across the entire lifecycle of medicinal products—from drug discovery to post-authorization [96]. The reflection paper outlines various AI applications, such as replacing animal models in preclinical development, optimizing patient selection in clinical trials, and enhancing post-authorization pharmacovigilance activities. However, it also highlights challenges like understanding algorithmic design, managing biases, and mitigating risks of technical failures. The EMA advocates for a human-centric approach to AI development and deployment, emphasizing compliance with existing legal requirements, ethical standards, and fundamental rights. Developers are encouraged to seek early regulatory support, particularly if AI/ML systems could impact the benefit-risk balance of a medicine [97].

Overall, both the FDA and EMA recognize the need for a balanced approach that fosters innovation in AI applications while ensuring patient safety, ethical use, and regulatory compliance. As AI technologies evolve, these regulatory bodies will likely continue to refine their frameworks and guidelines to address emerging challenges and ensure the responsible use of AI in drug development and clinical trials.

## 5. A Global Regulatory Landscape: EU vs. US and Industry Initiatives

AI represents an emerging and transformative force in global healthcare, yet it still operates in a landscape lacking a unified international legal and regulatory framework. The Global Initiative on Ethics of Autonomous and Intelligent Systems [98] aims to establish a comprehensive set of standards and principles designed to ensure that such AI systems are developed securely, ethically, and beneficial to society. This initiative also seeks to engage the public in formulating ethical frameworks, thereby enhancing societal understanding and awareness of the ethical issues associated with these technologies. By fostering public participation, the initiative encourages a more inclusive approach to addressing autonomous and intelligent systems’ moral, social, and economic implications, ensuring that these technologies serve the common good and reflect diverse societal values and perspectives [99].

While AI offers immense potential, its rapid development has raised concerns about its ethical and societal implications. As a result, governments worldwide are grappling with how to regulate this powerful technology. The European Union (EU) and the United States (US) have taken distinct approaches, each with strengths and weaknesses. EU law could guide the WHO in reforming the International Health Regulations (IHR). The EU has adopted a risk-based approach to AI regulation, embodied in the AI Act. This framework categorizes AI systems based on their potential risks to individuals and society. High-risk AI systems, such as those used in critical infrastructure or law enforcement, face stricter requirements, including mandatory human oversight and transparency. In contrast, the US has taken a more sector-based approach, relying on existing regulations in areas like healthcare, finance, and transportation to address AI-related concerns. This approach offers flexibility but can also lead to fragmentation and inconsistencies.

Beyond government regulation, individual companies and industry associations have also taken steps to address AI-related risks. Microsoft, for example, has developed its own internal AI principles to guide its research and development. However, there is a growing recognition that broader, industry-wide initiatives are needed to establish common guidelines and principles. Organizations should develop internal standards and guidelines aligning with ethical principles and regulatory requirements. These standards should cover various aspects of AI development and use, including data governance, risk assessment, and transparency. By establishing clear internal rules, organizations can ensure that their AI activities are conducted ethically and responsibly.

The EU’s AI Act establishes a robust enforcement mechanism, with significant fines for non-compliance. While lacking a centralized regulatory body, the US relies on a combination of federal agencies and industry self-regulation to oversee AI development and deployment.

The rapid pace of AI development and its global reach necessitate international co-operation. While the EU and US have taken different approaches, a growing consensus exists on the need for global standards and best practices. International organizations like the Organization for Economic Co-operation and Development (OECD) [15] and the Group of Seven (G7) promote responsible AI development and address shared challenges. While the EU and US have adopted distinct approaches, both recognize the importance of balancing innovation with ethical considerations. As AI advances, it will be crucial for governments, industry, and civil society to work together to ensure that this powerful technology is developed and used responsibly for all benefit.

### 5.1. Comparative Perspective on AI in Clinical Manufacturing and Commercialization

When comparing the FDA’s guidance on the use of AI in clinical trials with that of the European Medicines Agency (EMA), several key differences and similarities emerge, reflecting their respective regulatory philosophies and priorities [19,35].

The FDA has taken a proactive approach in addressing the integration of AI in clinical trials, recognizing the potential of AI to enhance drug development and patient outcomes. However, the agency’s guidance has often been criticized for being broad and high-level, focusing more on outlining expectations and concerns rather than offering specific, practical instructions for implementation. While encouraging innovation, this approach has left some industry stakeholders seeking more concrete direction on complying with regulatory standards when utilizing AI in clinical research. The FDA has also emphasized the importance of data quality, transparency, and the need for AI systems to be interpretable, ensuring that the technology’s use does not compromise patient safety or trial integrity.

On the other hand, the EMA has been somewhat more cautious in its approach to AI in clinical trials. The EMA’s guidance tends to be more detailed and prescriptive, ensuring that AI applications in clinical trials adhere to stringent data protection and ethical standards, particularly in the context of the General Data Protection Regulation (GDPR). The EMA has emphasized the importance of maintaining patient data privacy, informed consent, and the ethical use of AI, with a strong focus on ensuring that AI systems do not introduce bias or compromise the validity of clinical trial results. The EMA also emphasizes the need for transparency and explainability of AI algorithms, ensuring that all stakeholders, including regulators, can understand how AI-driven decisions are made.

The FDA’s guidance is generally more high-level and flexible, allowing for a broader interpretation of how AI can be integrated into clinical trials. In contrast, the EMA’s guidance is more detailed, focusing more on specific regulatory compliance, particularly regarding data protection under GDPR.

The FDA and EMA prioritize transparency and explainability in AI systems, but the EMA places a heavier emphasis on these aspects, especially regarding ethical considerations and patient safety. The opaque nature of many AI models, particularly those based on deep learning, makes it challenging for regulators to assess and verify the decision-making processes. The EMA has stated that transparent AI systems should be preferred, emphasizing the need for companies to invest in explainable AI technologies and maintain detailed documentation of AI decision-making processes.

Regarding data integrity and privacy, the EMA’s guidance is more stringent due to GDPR requirements, while the FDA has a slightly different focus despite similar concerns. Both agencies recognize the importance of data reliability and accuracy, and manufacturers should establish procedures for data collection and selection. Companies should consider improving traditional tools like encryption, access controls, and audit trails to ensure data integrity and handle more extensive and complex datasets. Investing in advanced cybersecurity measures and establishing rigorous data governance protocols is also essential.

Manufacturers must validate critical aspects of their operations through product and process lifecycle validation. AI/ML-specific features, such as continuous learning, pose challenges as AI systems evolve based on new data. We anticipate regulators will update their frameworks to encompass continuous monitoring and revalidation protocols for AI systems. Companies should implement robust change control systems to manage updates to AI algorithms and consider developing validation protocols that define objectives, scope, and acceptance criteria. These may include performance testing, comparison against reference methods, evaluation of algorithm robustness, and maintaining detailed records of algorithmic changes and performance metrics.

Using AI brings new and unknown risks, such as unfair or unreliable results due to untested algorithms. Manufacturers should implement thorough risk assessment procedures, adopt specific controls, enhance cybersecurity measures, and establish contingency plans for system failures.

While both the FDA and EMA recognize the transformative potential of AI in clinical trials, their approaches differ in terms of the level of detail, focus on data privacy, and the provision of practical guidance. The FDA’s more flexible, high-level guidance contrasts with the EMA’s detailed, prescriptive approach, reflecting the U.S. and Europe’s different regulatory landscapes and priorities. Understanding these differences is crucial for companies operating in both regions to navigate the regulatory environment and successfully integrate AI into clinical trials.

### 5.2. WHO Guidelines and Perspectives on AI

The WHO has released multilateral reports in 2021 and 2023 emphasizing the need for ethical and responsible use of AI in healthcare. These reports advocate for AI systems that uphold human dignity, equity, fairness, and accountability while highlighting significant legal and ethical challenges. The WHO highlights the need to ensure AI systems are safe and effective, quickly provide these systems to those in need, and encourage dialogue among all stakeholders, including developers, regulators, manufacturers, health workers, and patients. A key issue identified is the lack of harmonized standards and coordination among countries and stakeholders, particularly concerning data privacy and governance, underscoring the need for global collaboration to ensure AI benefits all fairly and inclusively [100,101].

The WHO guidelines on AI in clinical trials, drug and biologics product development, and manufacturing emphasize the importance of ethical considerations, data privacy, and addressing biases to prevent health disparities. AI should augment rather than replace human decision-making, and robust validation is crucial to ensure AI systems are accurate, reliable, and meet safety and efficacy standards. Transparency in AI algorithms, with clear documentation of their development and usage, is strongly advocated. In manufacturing, WHO recommends that AI be integrated within existing Good Manufacturing Practices (GMP), ensuring it enhances rather than compromises product quality, with continuous monitoring to detect and resolve any issues. Compared to the FDA and EMA, the WHO’s guidelines align closely, with all three organizations emphasizing ethics, validation, transparency, and the integration of AI within existing regulatory frameworks. However, the specific approaches and requirements differ slightly, reflecting each organization’s regulatory context.

The FDA and EMA provide comprehensive guidelines for integrating AI in clinical trials, drug and biologics product development, and manufacturing processes, emphasizing ethics, validation, and transparency. The FDA’s guidelines specifically focus on the validation and verification of AI algorithms to ensure they meet clinical decision support standards, and it integrates AI into GMP frameworks to maintain human oversight in manufacturing. Similarly, the EMA aligns its guidance with WHO’s principles, stressing the importance of rigorous validation, documentation, and continuous monitoring of AI systems to ensure accuracy, reliability, and safety in drug development and manufacturing. While both agencies emphasize ethical considerations and robust validation, their guidelines reflect nuanced differences due to their distinct regulatory environments. Many AI applications and methods do not significantly affect how current regulations or policies are interpreted or applied, making them largely policy agnostic. However, the advancements toward achieving parity between machine processing and human cognition have revealed instances where existing public policies fall short in addressing societal challenges, a phenomenon known as regulatory gaps.

Despite its expertise and constitutional mandate to regulate global health, the WHO’s role in setting new norms is limited to issuing non-binding guidelines and recommendations. Although these “soft” standards are influential and can shape national legislation and regulations, they fail to create enforceable legal norms. In contrast, the International Health Regulations (IHR) are an essential advancement in international law, providing a more formal framework for global health governance. Since states have a general obligation to co-operate under the UN Charter, including in health matters, the WHO should be empowered with enforcement authority to ensure compliance with the International Health Regulations (IHR), which should be amended to address the integration of AI in healthcare.

The current legal framework reveals the limitations of global public health and the relatively constrained role of the WHO despite its historical regulatory efforts. With the rise of international tech companies and evolving healthcare technologies, there is a pressing need for a new paradigm to address these global expansions. The WHO must enhance its authority and normative powers to address emerging issues related to AI in healthcare. AI can improve healthcare access and strengthen health systems, particularly in developing countries. However, challenges such as bias, data protection, and explainability must be addressed [35]. European regulations like GDPR, the Data Act, and the AI Act offer robust frameworks for ethical AI use [16]. WHO members need to collaborate on developing new, legally binding guidelines under the International Health Regulations (IHR) to ensure effective and responsible AI integration in healthcare.

### 5.3. Integration and Regulation of AL/ML in Pharmaceutical Manufacturing

Integrating AI and ML technologies into pharmaceutical manufacturing has introduced significant advancements in quality control and operational efficiency. The rapid rise and expansion of biologics, including living drugs such as cell and gene therapy (CGT), presents additional opportunities and challenges in this area. For example, manufacturing engineered T-cells, a promising cancer therapy, faces unique challenges due to their complexity and regulatory requirements [102]. These products face lengthy approval times in both the US and EU, with the timeline expected to worsen due to a technological shift in CGT drugs, especially for cancer. Using mathematical modeling and AI, we can simulate product behaviors, quantify the relationship between product attributes, patient physiology, and clinical outcomes, optimize treatments, and accelerate personalized medicine development [103]. As the field grows, regulatory frameworks must continuously adapt to manage emerging technologies effectively, balancing risks, benefits, and unique characteristics for traditional biologics and advanced medicinal product manufacturing. Although several guidelines exist for establishing these programs, they do not address the specific challenges of manufacturing CGT products or offer real-world evidence of the program’s effectiveness. In a GxP-regulated environment—including that regulating CGT, where Good Practice guidelines are essential to ensure product quality and patient safety—AI/ML tools have demonstrated their ability to enhance compliance by automating critical tasks and reducing human error. For instance, AI systems can scrutinize every step of the manual vial-filling process in aseptic processing, analyzing each segment to detect potential contamination risks. Similarly, AI introduces precision through image recognition technologies in environmental monitoring, moving beyond the traditional manual counting of microbial colonies [104]. In quality control testing, AI is now being used to automate the integration of chromatographic peaks and the visual inspection of injectable drugs, ensuring adherence to stringent regulatory standards [105].

AI’s impact extends beyond routine manufacturing processes to producing advanced therapy medicinal products (ATMP), medicines based on genes, tissue, cells, or combination, such as CGT. Its ability to automate critical steps in the manufacturing process improves the scalability and reliability of treatments, which is particularly beneficial in hospital settings, making personalized medicine more accessible and practical. However, adopting AI/ML in GxP environments requires careful consideration of regulatory requirements and validation processes.

As regulatory agencies work to develop and update guidelines that address the unique challenges posed by AI/ML, particularly in the context of generative AI, stakeholders must ensure that AI-driven processes comply with current Good Manufacturing Practices (GMP) and other GxP standards. Efforts to harmonize AI-related regulations across global platforms, such as those by the International Society for Pharmaceutical Engineering (ISPE) and the International Council on Harmonization (ICH), aim to reduce complexity for multinational companies and enhance operational efficiencies while maintaining compliance. The evolving regulatory landscape underscores the need for clear guidance on AI applications in Chemistry, Manufacturing, and Controls (CMC), process control, and quality assurance, ensuring these technologies can be safely integrated into pharmaceutical production [105].

Regulators are already gathering experience with AI/ML applications in the pharmaceutical industry. For example, the FDA has published a white paper describing how various centers, including CBER, CDER, CDRH, and OCP, collaborate to safeguard public health while fostering responsible and ethical innovation. The EMA has established a Quality Innovation Group (QIG) to support innovative medicine design, manufacturing, and quality control approaches. Both agencies are updating or creating new guidelines to align with the digital age, such as the FDA’s draft guidance on “Computer Software Assurance for Production and Quality System Software” and the EMA’s concept paper for revising GMP Annex 11 (computerized systems) [23]. Despite these efforts, the regulatory framework has not kept pace with the rapidly advancing field of AI/ML applications in GMP settings, especially in the pharmaceutical context, creating a need for continuous monitoring and adaptation.

The FDA and EMA also focus on specific guidance for the life sciences sector. The EMA has issued a draft reflection paper on using AI in the drug life cycle, which includes recommendations for model development and performance assessment based on quality risk management principles and ICH Q8, Q9, and Q10 guidelines. The FDA has initiated discussions with stakeholders to gather feedback on areas requiring clarity, such as regulatory oversight of AI in drug manufacturing and standards for developing and validating AI models used for process control [19,20,105,106].

The key elements of AI regulation in clinical trials, drug development, and manufacturing, as outlined by the FDA, EU, and WHO, are summarized in Table 3. Each organization has a unique approach, with the FDA being more flexible, the EU focusing on detailed regulations, and the WHO offering broad ethical guidelines. As AI continues to evolve, its applications in pharmaceutical manufacturing will require ongoing regulatory attention and adaptation. Engaging with regulators early and shaping these evolving guidelines will be crucial for stakeholders to navigate the complexities and harness the full potential of AI in this highly regulated industry.

#### 5.3.1. Digital Twins and Predictive Modeling

The pharmaceutical industry is increasingly adopting AI to enhance various stages of drug development, from discovery to post-marketing surveillance. Digital twins represent a cutting-edge application of AI in manufacturing process control by creating virtual simulations of physical systems based on real-time sensor data [5,107]. A DT is a virtual representation of a real-world object or system designed to reflect its behavior in real time and continuously updated based on historical data. Unlike traditional simulations, DTs run in parallel with their real-world counterparts and can simultaneously simulate multiple processes, allowing for enhanced process optimization and development. Advanced cloud technologies support DTs in managing highly controlled industrial environments, facilitating continuous data exchange and self-monitoring. This multidirectional information flow enables DTs to provide comprehensive and accurate representations of a system’s status, predict outcomes, suggest necessary actions, and even support closed-loop process control, making them more effective than classical simulations [108]. A key advantage of AI-driven models is their ability to perform counterfactual analyses, which involve rapidly simulating hypothetical scenarios to predict possible outcomes. This capability allows pharmaceutical companies to conduct virtual experiments, explore different manufacturing conditions, and understand the potential impacts of various interventions without requiring extensive physical trials. By simulating “what-if” scenarios, these models can identify the optimal process parameters that minimize waste, reduce costs, and improve overall efficiency.

Furthermore, AI models enhance decision-making by integrating vast amounts of data from diverse sources, such as sensor networks and historical databases, enabling a more holistic and data-driven approach to process optimization [108]. Critical challenges in applying AI in biopharmaceutical manufacturing include the absence of clear regulatory guidelines, gaps in data completeness, complexities in conducting risk assessments, a lack of specialized tools tailored for biopharma, and a shortage of skilled professionals. These issues are compounded by the rapidly evolving landscape of AI technologies, which demands continuous updates to regulatory frameworks and increased training for personnel to utilize AI tools [109] effectively.

Integrating DT technology early in product development, with a phase-appropriate and change management approach, enables pharmaceuticals to adopt faster processes, achieve higher efficiencies, and enhance customer engagement. However, bias risks can be more pronounced in allogeneic therapies, mainly when using digital twins to predict cell quality in treatments like CAR T cell therapies. Digital twins are virtual models based on real-time data, often using data from healthy donors to predict outcomes. This reliance on population-based rather than patient-specific data can introduce biases, creating synthetic datasets that may not represent the intended treatment population. As DTs and patient-specific data drive advancements in clinical trials, they accelerate ATMP/CGT product development processes such as safety and efficacy assessments, improve outcomes, and reduce costs.

A key example is in silico clinical trials for TCR-engineered T-cell therapies for cancer. Using a quantitative system pharmacology model, patient-specific digital twins were developed to stimulate T cell kinetics, created for patients with metastatic HPV-associated cancers, and identified stem cell-like memory T cells (Tscm) as crucial for persistence and functional outcomes. Simulating the in-silico trials, the model predicted that enriching Tscm in the infused product could improve persistence and allow for lower dosing [110]. This application illustrates how digital twins can optimize therapeutic strategies, reduce variability, and accelerate drug development. DT models face significant challenges, particularly regarding the availability of clinical data necessary for algorithmically generating clinical decision support and trust reliability of the treatment options evaluated [111,112,113]. To tackle these issues, a collaborative approach can effectively mitigate the regulatory and ethical concerns of integrating AI-based decision-making tools into clinical practices. This strategy emphasizes the need for high-quality data, effective historical data management and awareness of synthetic biases, and thorough documentation and evaluation of model validation to ensure effectiveness and equitable outcomes in drug development.

In biomanufacturing, AI-driven digital twins are precious for optimizing complex and sensitive processes [114]. For example, these tools can precisely control bioreactor conditions by continuously analyzing data streams related to temperature, pH, and nutrient levels. The models can predict the impact of slight variations on the quality and yield of the biological product, allowing for real-time adjustments that maintain optimal production conditions [115]. This intelligent closed-loop control ensures consistent product quality and significantly reduces the likelihood of batch failures, which can be costly and time-consuming. By providing real-time insights and recommendations, AI-driven digital twins enable pharmaceutical manufacturers to maintain high precision and reliability in their operations [116].

Additionally, integrating AI in biomanufacturing fosters innovation by accelerating the development of new therapies and enhancing the scalability of production processes. AI models can analyze patterns and trends in vast datasets to identify novel drug candidates, optimize formulation compositions, and predict how changes in the manufacturing process could affect drug efficacy and safety. This capability is critical as the industry shifts towards more personalized medicine approaches, where small-batch production and rapid adjustments to manufacturing processes are crucial. By optimizing these processes, AI helps bring new therapies to market faster and supports the production of complex biologics tailored to individual patient needs [113]. Integrating AI and digital twins into the pharmaceutical industry revolutionizes how complex data are handled, analyzed, and utilized. It enables more precise and efficient manufacturing processes, reduces time-to-market for new drugs, and ultimately leads to better patient outcomes. As AI technology advances, its role in the pharmaceutical landscape is expected to expand, driving further innovations and setting new standards for drug development and production [117].

#### 5.3.2. Emerging AI-Focused Standards for Advanced Manufacturing Technologies

Integrating AI into advanced manufacturing technologies (AMT) has transformed healthcare drug and biological product development. AI enhances precision, efficiency, and adaptability, addressing critical challenges in producing complex therapeutics such as biologics, gene therapies, and personalized medicine. Regulatory bodies, including the FDA and organizations like the NIST, play a crucial role in guiding the effective and safe implementation of AI in these processes [118].

AI algorithms are increasingly employed to monitor and optimize manufacturing processes in real-time. Continuous data collection and analysis enable AI to predict potential deviations and adjust parameters to maintain optimal conditions, ensuring consistent product quality. This capability is essential in biologics production, where even minor process variations can significantly affect product efficacy and safety. The FDA’s guidelines stress the importance of AI-driven process control, advocating for AI to complement existing technologies such as Process Analytical Technology (PAT) and continuous manufacturing systems [73]. AI also facilitates the transition from traditional batch manufacturing to constant manufacturing—a method endorsed by the FDA for its efficiency and flexibility. Continuous manufacturing allows uninterrupted production, reducing lead times and minimizing human error. AI systems are integral to monitoring and controlling material flow, and maintaining quality throughout the process. The FDA’s guidance highlights AI’s role in enabling rapid scaling to meet market demands, particularly for vaccines and other time-sensitive therapies [119].

The ability of AI to analyze extensive datasets enhances the sophistication of quality assurance measures. Machine learning models can identify patterns and anomalies that traditional methods might overlook, allowing for early detection of potential quality issues. The FDA and NIST emphasize AI’s role in quality assurance, especially in preventing defects and ensuring compliance with regulatory standards. Additionally, AI is pivotal in predictive maintenance, analyzing equipment data to forecast and prevent failures, thus minimizing downtime and maintaining operational efficiency. Adherence to regulatory standards is critical in the highly regulated drug and biological product manufacturing field. AI can streamline documentation processes, ensuring all manufacturing steps are accurately recorded in real-time. This digital traceability simplifies audits and provides a robust framework for demonstrating compliance with regulations from agencies like the FDA and the European Medicines Agency (EMA). AI-driven systems also aid in preparing regulatory submissions by automatically generating reports that meet required data standards and formatting. AI is incredibly transformative in producing personalized medicine and advanced therapeutics, such as CAR-T cell therapies and gene editing technologies. These treatments demand highly specialized manufacturing processes tailored to individual patients. AI helps manage the complexity of these processes, ensuring that each therapeutic product meets stringent quality and safety standards. The FDA’s guidelines on advanced therapeutics underscore AI’s role in enabling scalable production of personalized treatments, thereby increasing accessibility to these advanced therapies. As AI technology continues to evolve, its integration into manufacturing processes is expected to deepen, leading to development of “smart” manufacturing environments. These environments will feature fully automated, AI-driven production lines capable of quickly adapting to new challenges and innovations. Collaborative efforts by the FDA, NIST, and the National Institute for Innovation in Manufacturing Biopharmaceuticals (NIIMBL) are crucial in shaping these advancements. NIST’s development of AI standards and performance metrics, combined with the FDA’s regulatory frameworks, will ensure that intelligent manufacturing in drug and biologic production adheres to the highest safety and quality standards.

The FDA has also addressed the potential of AI/ML to enhance pharmaceutical manufacturing, as highlighted in its Second Discussion Paper. This paper elaborates on how advanced analytics using AI/ML can support various aspects of manufacturing, including process controls, equipment reliability, and early warning systems for process deviations. The FDA identifies four critical areas for AI/ML applications: process design optimization, advanced process control implementation, intelligent monitoring and maintenance, and trending activities. The FDA acknowledges the need for robust standards for trustworthy AI, focusing on characteristics such as explainability, reliability, privacy, safety, security, and bias mitigation [119]. The agency seeks feedback from stakeholders to refine these standards and ensure they address specific concerns in drug development, including governance, data quality, and model performance. While AI and ML hold immense potential for revolutionizing drug development and manufacturing, realizing this potential requires a balanced approach to regulatory compliance and ethical standards. Addressing these challenges head-on, coupled with global harmonization efforts, will enable the industry to harness the benefits of AI while maintaining the highest standards of data protection and patient care.

#### 5.3.3. AI-Enhanced Manufacturing Processes Monitoring

AI technologies rapidly advance in pharmaceutical manufacturing, offering significant potential to enhance process monitoring, quality assurance, and operational efficiency. NIST has been at the forefront of exploring AI-enhanced monitoring to optimize manufacturing processes, providing real-time insights and predictive analytics that traditional methods could not achieve. AI systems can analyze vast amounts of data from sensors and control systems, enabling continuous real-time monitoring and adjustments to critical parameters. This is crucial for maintaining quality and compliance in pharmaceutical production.

To further advance the integration of AI in manufacturing, NIST announced plans to establish a Manufacturing USA institute in Spring 2024. The agency aims to select an applicant team “most capable of establishing and leading a Manufacturing USA institute to accelerate the use of AI for strengthening the resilience of manufacturing processes for the nation’s manufacturers”. This initiative encourages collaboration among industry stakeholders, academia, federal laboratories, and state and local governments, ensuring a broad base of expertise and resources to drive innovation in AI applications. A key component of AI-focused manufacturing process monitoring is testing and validating new data streams and system enhancements in a controlled, low-risk environment. Platforms like CROW (Cyber-physical Research on Working setups) provide a benchtop setup where pharmaceutical manufacturers can evaluate various AI tools and systems without fearing losing time or resources in a full-scale production facility. This “try before you buy” philosophy is crucial for facilities with limited resources, allowing them to invest confidently in technologies that promise the highest returns. Experiments conducted on setups like CROW enable developers to test and benchmark AI-based products and allow manufacturers to better understand these products’ effects, compare solutions, and identify potential pain points. These efforts also help develop best practice guides and standard operating procedures (SOPs) to manage, maintain, and sustain intelligent automation now and in the future. Specific areas targeted by AI-enhanced monitoring efforts include:

- Implementation of manufacturing data exchange standards;
- Cybersecurity monitoring to safeguard network and information integrity;
- Digital twin or digital surrogate simulations for process testing and control;
- Reliability, prognostics, and health management of manufacturing equipment;
- Product quality monitoring to ensure compliance with regulatory standards;
- System-level evaluations to assess overall process efficiency and effectiveness;
- Human interactivity and feedback mechanisms through natural language processing;
- Trust and trustworthiness requirements for AI systems.

Future initiatives also anticipate enhancements in robot and co-bot control, advanced material handling, standardization and implementation of data exchange protocols, and digital thread mapping and utilization. By leveraging these advanced monitoring capabilities, pharmaceutical manufacturers can detect anomalies or deviations from standard operating procedures more quickly than human inspectors, reducing waste and preventing costly recalls or compliance violations [120].

Moreover, AI-enhanced monitoring supports predictive maintenance strategies by anticipating equipment failures before they occur, minimizing downtime, and extending the lifecycle of critical manufacturing equipment. In a highly regulated environment, such as pharmaceutical manufacturing, these capabilities help maintain compliance with GMP by providing continuous oversight and control over the production environment. As regulatory bodies continue to develop guidelines for AI applications in manufacturing, the pharmaceutical industry must engage in collaborative efforts, such as those encouraged by NIST’s upcoming Manufacturing USA institute. This collaboration will be crucial in establishing best practices and standards for AI-enhanced monitoring technologies, ensuring these tools are effective and compliant with regulatory requirements [120]. Proactively integrating AI technologies into their monitoring systems, pharmaceutical manufacturers can enhance their process monitoring capabilities and maintain a competitive edge in a rapidly evolving industry [121].

## 6. Conclusions and Path Forward

Integrating AI and ML in drug development and clinical trials presents a double-edged sword, offering transformative potential while posing significant challenges. On one hand, AI technologies promise to enhance operational efficiency, improve trial design, and accelerate the development of new therapies. On the other hand, they introduce ethical, regulatory, and data protection challenges that must be carefully managed to ensure patient safety and data integrity. Rules-based AI, operating on predefined rules and requirements, provides stability and transparency but is limited by the knowledge and capabilities of its creators. In contrast, data-driven responsible AI, such as machine learning algorithms, adapts and learns from data, offering flexibility and the ability to handle complex scenarios. However, their effectiveness depends on the quality of data and the system’s ability to manage and interpret it correctly. Misapplications or data issues can lead to poor results, underscoring the need for rigorous testing and validation. Using a risk assessment framework in model development helps determine the appropriate balance between automation and human oversight, enhancing safety and overall system performance.

The FDA and EMA have proactively addressed these challenges, highlighting the importance of collaboration among regulators, industry stakeholders, and data protection experts. Regulators are adopting adaptive frameworks to address the dynamic nature of AI models. These frameworks emphasize ethical principles such as fairness, accountability, transparency, and inclusivity in oversight. Integrating Real-World Evidence (RWE) powered by AI tools is increasingly accepted in regulatory submissions. Additionally, regulators are expected to scrutinize data integrity, bias mitigation strategies, and the explainability of AI systems during inspections. These represent future trends in AI regulations for clinical trials and drug development, highlighting the evolving landscape of regulatory expectations (Figure 4). The recent FDA workshop emphasized the need to manage biases, ensure data quality, and maintain rigorous validation and transparency of AI algorithms. Continuous monitoring and adaptation are essential to navigate the evolving landscape of AI in drug development, along with robust regulatory frameworks that prioritize ethical considerations related to data integrity, privacy, and risk-based decision-making (Figure 4).

The integration of AI within life sciences and drug development is set to redefine how therapies are designed, tested, and personalized. Particularly in cell and gene therapy, AI is an essential tool for enhancing precision and efficiency while preserving the critical judgment of researchers, manufacturers, and clinicians. AI is becoming a key driver in tackling rare diseases and personalizing treatments by processing complex datasets and evaluating multiple variables simultaneously.

Key areas of focus moving forward should include the following:

- Personalization: Leveraging AI to advance personalized medicine, ensuring treatments are tailored to individual patient profiles.
- Regulatory Frameworks: Developing and refining robust AI validation and monitoring frameworks to ensure compliance and safety.
- Ethical Considerations: Addressing data privacy, security, and decision-making issues to maintain public trust and uphold patient rights.

NIST has been instrumental in developing testing methods and metrics to differentiate useful AI tools from ineffective ones, emphasizing the importance of good data and community feedback in this process. Our regulatory frameworks must adapt as AI technologies evolve to ensure innovation is pursued ethically and with patient safety at the forefront.

True precision in medicine, once currently limited to adapting therapeutic protocols or crafting ATMPs for individual patients, is advancing with AI-driven methods development and validations. These approaches enable personalized predictions for CAR-T/NK therapy outcomes, including response rates, toxicity risks like cytokine release syndrome, and neurotoxicity. Furthermore, AI-driven bioinformatics facilitates identifying patient-specific mutations, advancing tumor profiling, neoantigen targeting, and the high-throughput design of personalized CAR-T/NK cells. The development of AI-based tools for molecular engineering, receptor design, and automation continues to optimize manufacturing processes, making personalized ATMPs a more achievable reality.

Regulatory frameworks face significant challenges in adapting to personalized ATMPs. Unlike traditional fixed-product approaches, personalized therapies require dynamic, patient-specific designs, prompting the need for flexible, responsive frameworks. Current guidelines from the FDA and EMA address AI’s role in precision medicine but have yet to incorporate individualized ATMP development fully. A combined approach will likely emerge to support AI’s safe and effective application in this transformative area, utilizing existing rigid frameworks and new AI-specific standards. This progress underscores international collaboration and innovation’s critical importance in aligning regulatory systems with the evolving technological landscape.

In conclusion, while AI and ML promise to revolutionize drug development and clinical trials, realizing this potential requires a balanced approach to regulatory risk compliance and ethical standards. By addressing these challenges directly and working towards global harmonization, the industry can foster cross-functional, multidisciplinary collaboration among stakeholders. This includes promoting knowledge sharing to break down silos, leveraging the expertise of domain specialists in AI augmentation, and ensuring the transparency of AI/ML models. Establishing a robust quality assurance program to mitigate the risks of training bias and data drifts is also essential. Together, these efforts will lead to safer and more effective applications of AI/ML in oncology labs and clinics while upholding the highest standards of data protection and patient care (Figure 4).

## Figures and Tables

**Table 1:** Executive Order on AI in drug development [36].

Enhance AI Utilization in Drug Development Goals: Improve Safety, Efficacy, Efficiency Focus Areas: Data Quality, Transparency, Risk Management		
FDA	NIST	Other Agencies
Ensure AI tools meet safety and efficacy standards Review AI in Drug Development Provide Guidelines for AI integration Risk Management and Post-Market Surveillance Stakeholder Engagement	Develop AI technology Standard and Benchmarks Promoting Performance metrics and Best Practices Provide Technical guidance Risk management framework Governance and Policy	Ethics and Privacy Data Protection Global Partnership International Coordination Innovative Support

**Table 2:** Summary of the FDA workshop highlights and takeaways.

Theme	Key Insights	Takeaways
Optimizing Model Design Through Multidisciplinary Expertise	Collaboration among computational scientists, clinicians, regulatory experts, and ethicists is critical. Ensures AI models are clinically relevant, technically robust, and compliant with regulations.	Multidisciplinary approaches ensure models align with clinical practices and regulatory needs, supporting better AI design and outcomes.
Using and Creating Data: Clinical Development and Analysis	AI aids in trial design, recruitment, and data analysis but depends heavily on data integrity. Emphasis on governance practices: standardization and harmonization. Use of single-cell data for in silico predictions and dose optimization.	Effective data management unlocks AI-driven insights, improves pharmacokinetics, and enhances precision in drug development. Clear alignment with FDA Good Clinical Practices is necessary.
Balancing Performance, Explainability, and Transparency	High-performing ’black box’ AI models need better explainability tools. Stakeholders must trust AI outputs for adoption. Proposals included dual-model systems for better interpretability.	Explainability is crucial for regulatory approval and clinical acceptance. Innovations in transparency tools can bridge the gap between performance and trust.
Identifying Gaps and Challenges	Global policy inconsistencies and lack of continuous AI validation post-market. Need for coordinated international pathways. Emphasis on risk-based approaches, real-world data integration, and guidelines for safety.	Harmonization of global policies and better guidelines can improve adoption and regulatory alignment. Focus on risk-based approaches for long-term validation.
Data Integrity and Quality Challenges	Standardized demographic and biomarker data is missing, limiting AI’s predictive power. Inconsistent definitions of race/ethnicity and lack of social determinants of health (SDoH) data. Need for globally representative datasets. Privacy/security remain critical.	Creating centralized repositories for biomarker data, addressing biases, and enhancing privacy protections will improve inclusivity and model performance across diverse populations.
AI Implementation in Drug Development	Scalable AI systems must comply with diverse global healthcare and regulatory systems. Human-led governance and transparency are essential. ICON’s Cassandra highlights AI’s success in regulatory efficiency and real-world evidence.	Robust model validation, transparency, and collaboration with regulatory bodies are essential for scaling AI systems across diverse markets.

**Table 3:** The key elements of AI regulation in clinical trials, drug development, and manufacturing outlined by the FDA, EU, and WHO.

Element/Agency	FDA (United States)—Refs. [19,20,21,32,33,48,49,50,51,52,56,60,65,91,99]	EU (European Union)—Refs. [17,23,31]	WHO (World Health Organization)—Refs. [92,93,94]
Regulatory Framework	Flexible, sector-based approach.	Risk-based approach embodied in the AI Act, categorizes AI systems based on risk.	Non-binding guidelines; emphasizes ethical AI use, safety, and effectiveness.
Ethical Considerations	Emphasizes ethics, transparency, and the need for interpretable AI systems.	Strong focus on ethics, patient data privacy (GDPR), and AI transparency.	Advocates for ethical AI systems that uphold human dignity, equity, fairness, and accountability.
AI in Clinical Trials	High-level guidance, focusing on expectations and data quality; AI systems should be interpretable and safe.	Detailed, prescriptive guidance; strong focus on data protection, patient privacy, and algorithm transparency.	It recommends that AI augment human decision-making and prioritize transparency and validation in clinical trials.
Data Privacy and Governance	Data reliability and accuracy; general concerns around data governance, cybersecurity, and continuous monitoring.	Stringent data protection under GDPR emphasizes transparency and risk-based compliance with data handling.	Emphasizes global collaboration on data governance; advocates for robust safeguards against biases and data protection.
AI System Validation	Focuses on validating and verifying AI algorithms, particularly for clinical decision support.	Strong focus on validation, continuous monitoring, and ensuring accuracy and reliability through detailed processes.	AI systems must meet safety and efficacy standards; continuous monitoring and validation are crucial in manufacturing.
Transparency and Explainability	Encourages transparency and interpretable AI systems, but with flexibility in implementation.	Heavy emphasis on explainable AI, ensuring stakeholders understand how AI-driven decisions are made.	Strong emphasis on transparent algorithms, with clear documentation of their development and usage.
Integration with Existing Frameworks	Encourages integration into Good Manufacturing Practices (GMP), maintaining human oversight in manufacturing.	It aligns with GMP frameworks and stresses rigorous documentation and continuous monitoring in clinical and manufacturing settings.	Recommends AI integration within existing GMP, ensuring enhanced product quality, with ongoing monitoring.
Risk Management and Cybersecurity	Emphasizes robust change control, cybersecurity, and risk assessments for AI in clinical trials and manufacturing.	Focuses on risk-based assessment and AI system security, incorporating strict data integrity and privacy controls.	Advocates for managing risks through robust cybersecurity, data integrity, and ethical considerations across AI systems.
Global Standards and Co-operation	Lacks unified international standards; focuses on federal agency and industry self-regulation.	Recognizes the need for global AI standards; supports international co-operation for AI governance.	Calls for global collaboration to address AI-related challenges, including data privacy, explainability, and fairness.
Enforcement Mechanisms	Relies on a combination of federal agencies and industry self-regulation; no centralized regulatory body.	Robust enforcement mechanism with significant fines for non-compliance with the AI Act.	Issues non-binding recommendations; limited enforcement capacity compared to regional regulatory bodies.
Role in Setting Norms	Regulates AI under existing frameworks, with no direct enforcement of global standards.	Acts as a leader in developing comprehensive AI regulations that could influence global standards.	Issues non-binding guidelines: has a limited role in enforcement but influences national and international regulations.
Focus on Continuous Monitoring	Encourages continuous learning in AI systems, especially in drug development and clinical trials.	It stresses the importance of ongoing monitoring, especially in AI systems used in clinical trials and manufacturing.	Emphasizes the need for continuous monitoring in AI systems to detect and resolve issues.
